# Dasatinib reduces FAK phosphorylation increasing the effects of RPI-1 inhibition in a RET/PTC1-expressing cell line

**DOI:** 10.1186/1476-4598-9-278

**Published:** 2010-10-18

**Authors:** Dario Caccia, Francesca Miccichè, Giuliana Cassinelli, Piera Mondellini, Patrizia Casalini, Italia Bongarzone

**Affiliations:** 1 Department of Experimental Oncology and Molecular Medicine, Proteomics Laboratory, Fondazione IRCCS Istituto Nazionale Tumori, Milan, Italy; 2 Department of Experimental Oncology and Molecular Medicine, Molecular Pharmacology Unit, Fondazione IRCCS Istituto Nazionale Tumori, Milan, Italy; 3 Department of Experimental Oncology and Molecular Medicine, Molecular Targeting Unit, Fondazione IRCCS Istituto Nazionale Tumori, Milan, Italy

## Abstract

**Background:**

TPC-1 is a papillary thyroid carcinoma (PTC)-derived cell line that spontaneously expresses the oncogene *RET/PTC1*. TPC-1 treated with the RET/PTC1 inhibitor RPI-1 displayed a cytostatic and reversible inhibition of cell proliferation and a strong activation of focal adhesion kinase (FAK). As dasatinib inhibition of Src results in reduction of FAK activation, we evaluated the effects of TPC-1 treatment with dasatinib in combination with RPI-1.

**Results:**

Dasatinib (100 nM) strongly reduced TPC-1 proliferation and induced marked changes in TPC-1 morphology. Cells appeared smaller and more contracted, with decreased cell spreading, due to the inhibition of phosphorylation of important cytoskeletal proteins (p130^CAS^, Crk, and paxillin) by dasatinib. The combination of RPI-1 with dasatinib demonstrated enhanced effects on cell proliferation (more than 80% reduction) and on the phosphotyrosine protein profile. In particular, RPI-1 reduced the phosphorylation of RET, MET, DCDB2, CTND1, and PLCγ, while dasatinib acted on the phosphorylation of EGFR, EPHA2, and DOK1. Moreover, dasatinib completely abrogated the phosphorylation of FAK at all tyrosine sites (Y576, Y577, Y861, Y925) with the exception of the autoactivation site (Y397). Notably, the pharmacological treatments induced an overexpression of integrin β1 (ITB1) that was correlated with a mild enhancement in phosphorylation of ERK1/2 and STAT3, known for their roles in prevention of apoptosis and in increase of proliferation and survival. A reduction in Akt, p38 and JNK1/2 activation was observed.

**Conclusions:**

All data demonstrate that the combination of the two drugs effectively reduced cell proliferation (by more than 80%), significantly decreased Tyr phosphorylation of almost all phosphorylable proteins, and altered the morphology of the cells, supporting high cytostatic effects. Following the combined treatment, cell survival pathways appeared to be mediated by STAT3 and ERK activities resulting from integrin clustering and FAK autophosphorylation. EphA2 may also contribute, at least in part, to integrin and FAK activation. In conclusion, these data implicate ITB1 and EphA2 as promising therapeutic targets in PTC.

## Background

The transformation of normal follicular thyroid cells into cancer cells is a multistep process involving genetic alterations associated with aberrant growth control, loss of differentiation, and invasiveness [1]. Thyroid carcinomas can be divided into four groups: papillary, follicular, medullary, and anaplastic carcinomas [2]. Papillary thyroid carcinoma (PTC) is the most prevalent of these cancer subtypes. PTC is associated with characteristic genetic alterations that include rearrangement of the tyrosine kinase receptor oncogenes *RET *and *NTRK1 *and point mutations in the *Ras *and *BRAF *genes [3,4]. Specific rearranged forms of *RET *were detected in PTC that are the result of double-stranded DNA breaks (mostly radiation-induced), leading to the erroneous reparative fusion of the 3' portion of *RET *to the 5' portion of a constitutively-expressed unrelated gene and producing *RET/PTC *genes [5]. Approximately 17 different hybrid oncogenes have been reported; the most prevalent variants are *RET/PTC1 *(the *H4-RET *fusion) and *RET/PTC3 *(the *RFG-RET *fusion), accounting for > 90% of all known rearrangements [6,7].

An increasing number of tyrosine kinase inhibitors of low molecular weight are being tested and used in clinical practice as anticancer agents [8]. For instance, PLX4032 is a highly-selective inhibitor of BRAF kinase activity with an IC_50 _of 44 nmol/l against the BRAF^V600E ^mutant [9], while RPI-1 is a selective inhibitor of RET kinase activity [10]. Specifically, RPI-1 is an orally-available, indolinone-based tyrosine kinase inhibitor, initially described as an inhibitor of the fusion protein RET/PTC1. RPI-1 showed high effectiveness in controlling the growth of thyroid tumors by inhibiting tyrosine kinase activity, expression, and signaling of RET in TT cell line [11]. Moreover, treatment with RPI-1 inhibited the proliferation of the TPC-1 cell line, which harbors the RET/PTC1 rearrangement, and induced accumulation of these cells at the G2 cell cycle phase. In treated cells, RET/PTC1 tyrosine phosphorylation was abolished along with its binding to Shc and phospholipase C, abrogating constitutive signaling mediated by the oncoprotein. Like many other inhibitors, RPI-1 causes a cytostatic and reversible inhibition of cell proliferation [12].

We have previously reported that thyroid tumor cell lines expressing RET oncoproteins after RPI-1 treatment maintained strong activation of focal adhesion kinase (FAK), one of the most prominent phosphorylated proteins in the PTC cell line TPC-1 [13]. FAK is a non-receptor tyrosine kinase that localizes at focal adhesions and is activated by integrin clustering or by growth factor receptor activation. FAK presents six phosphorylation sites that are differentially phosphorylated in response to various stimuli. After autophosphorylation at Y397, FAK becomes activated and can recruit other proteins. The binding of Src leads to the full activation of FAK through the Src-mediated phosphorylation of Y576 and Y577 in the FAK activation loop [14].

Src family kinases (SFKs) represent the largest family of non-receptor tyrosine kinases that interact directly with receptor tyrosine kinases, signal transducers, activators of transcription, and molecules involved in cell adhesion and migration. Aberrant expression or activation of SFKs causes perturbations in these activities, leading to transformation and progression of malignant disease [15]. Moreover, Src activity contributes to the growth and survival of malignant cancers such as breast, prostate, and lung, implicating this kinase as a promising therapeutic target for a wide range of human cancers [16,17].

Dasatinib is an ATP-competitive tyrosine kinase inhibitor that inhibits all SFKs at low concentrations (IC_50 _< 1.0 nM); dasatinib is currently approved for imatinib-resistant/intolerant BCR-ABL+ leukemias. At higher concentrations, dasatinib may inhibit other tyrosine kinases such as p38, Akt, FAK, and others [18]. This drug has demonstrated antiproliferative effects on lung and prostate tumor cell lines [16,17], and its effects on breast cancer are currently under investigation [19]. Accordingly, we wished to investigate the effects induced by dasatinib, alone or in combination with RPI-1, on the TPC-1 cell line, by evaluating cell proliferation, morphological changes, and phosphorylation reduction. Additionally, we examined the mechanisms that permit TPC-1 survival following treatment with the combination of the two drugs.

## Results

### Effect of dasatinib on TPC-1 cells

**Figure 1:**
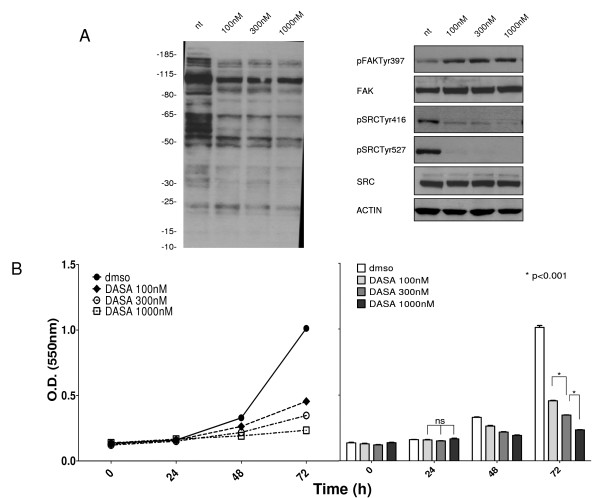
**Effect of dasatinib on TPC-1 cells**. (A) *Left*, phosphotyrosine protein profiles of TPC-1 cells treated with various dasatinib concentrations (100, 300, and 1000 nM). *Right*, Src and FAK phosphorylation status following dasatinib treatment. Results were normalized by comparison with beta-actin. (B) Proliferation assay. *Left*, proliferation curves of TPC-1 cells treated with 100 nM, 300 nM, and 1000 nM dasatinib after 24, 48, and 72 hours. *Right*, statistical visualization of the proliferation curves. After 72 hours, the TPC-1 cell proliferation was significantly (p < 0.001) reduced by 60%, 65%, and 75% after treatment with 100 nM, 300 nM, and 1000 nM dasatinib, respectively.

TPC-1 cells, grown in DMEM supplemented with 10% calf serum, were treated with various concentrations of dasatinib (100, 300, and 1000 nM) in order to evaluate Src inhibition and reduction of cell proliferation. As shown in Figure 1A, the phosphotyrosine profiles of cells treated with the three different dasatinib concentrations did not change. Src phosphorylation at Y416 was reduced at all dasatinib concentrations, and the regulatory site of SRC (Y527) was switched off while the autophosphorylation of FAK Y397 appeared to be enhanced. The proliferation assay (Figure 1B) highlighted an association between reduction in proliferation and increase in dasatinib concentration. In particular, proliferation was reduced approximately 60%, 65%, and 75% following 72 hours of treatment with 100 nM, 300 nM, and 1000 nM of dasatinib, respectively.

### Dasatinib-induced morphological changes

**Figure 2:**
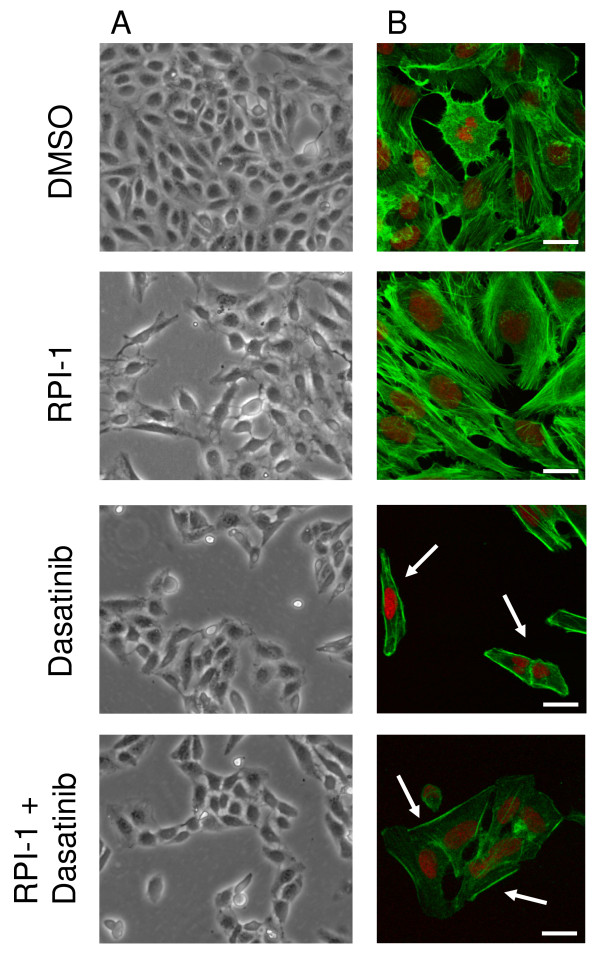
**Morphological effects of drug treatments**. (A) Optical microscope images of TPC-1 cells before and after drug treatments. Cell spreading increased following RPI-1 treatment. Dasatinib treatment changed cellular morphology, characterized by a smaller appearance. (B) Confocal microscope images of TPC-1 cells before and after drug treatments. The cells were stained with phalloidin and DRAQ5. The actin cytoskeletal structure of the cells was modified following dasatinib treatment; the branching actin structures appeared to be thickened at the cell edges (white arrows). Confocal images (512 × 512 pixels) were obtained using a 60× oil immersion lens and were analyzed using ImagePro 6.3 software. Scale bars, 40 μm.

The mesenchymal-like TPC-1 cells normally grow in a flattened pattern, characterized by many filopodia-like processes. RPI-1 treatment produced a marked enlargement and flattening of the cellular morphology and an increase in cell spreading (Figure 2A) [12]. Moreover, an increase in actin stress fibers was observed following RPI-1 treatment. After dasatinib treatment, the cells displayed a very different morphology that was characterized by a smaller and contracted appearance, and decreased cell spreading was observed (Figure 2A). As revealed by confocal microscopy, the actin cytoskeleton of the cells was modified, creating a more compact cell body in which the branching actin structures appeared to be thickened on the cell edges (white arrows Figure 2B).

**Figure 3:**
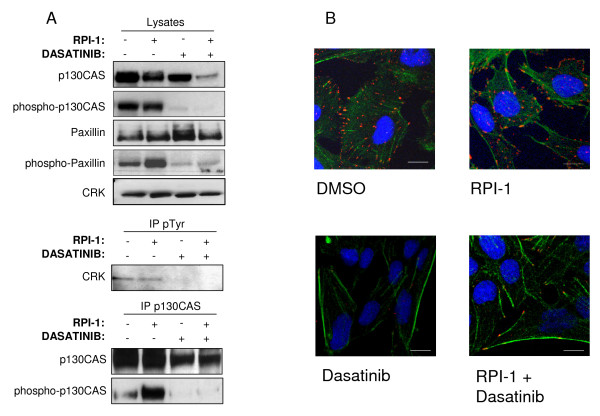
**Cytoskeletal and focal adhesion changes**. (A) Phosphorylation status of the cytoskeletal regulatory proteins p130^CAS^, Crk, and paxillin following drug treatments. Immunoblot analysis indicates down-modulation of cytoskeletal regulatory proteins in terms of protein levels (only p130^CAS^) and tyrosine phosphorylation following dasatinib treatment. (B) Confocal microscope images of TPC-1 cells before and after drug treatments. Cells were stained for phospho-paxillin (red), actin (green phalloidin) and DRAQ5 to highlight the focal adhesions. In control and RPI-1-treated cells, phospho-paxillin accumulated in well-defined zones around the focal adhesions, while after dasatinib treatment, phospho-paxillin disappeared. Confocal images (512 × 512 pixels) were obtained using a 60× oil immersion lens and analyzed using ImagePro 6.3 software. Scale bars, 10 μm.

To further investigate the morphological effects induced by dasatinib, we probed the protein lysates of the treated cells for phosphorylation of the cytoskeletal regulatory proteins p130^CAS^, Crk, and paxillin, all of which are substrates of SFKs. We also analyzed the cellular distribution of the phosphorylated form of paxillin, a focal adhesion docking protein. After dasatinib treatment we observed inhibition of Crk phosphorylation and a consistent reduction in the phosphorylation of p130^CAS ^and paxillin (Figure 3A). Since the combined treatment reduced the total level of p130^CAS ^protein, we analyzed the phosphorylation of p130^CAS ^before and after immunoprecipitation with anti-p130^CAS ^antibody, confirming the reduction in protein phosphorylation (Figure 3A and Additional file 1: Immunoblot analysis of p130^CAS ^immunoprecipitation). In the control and the RPI-1-treated cells, phospho-paxillin accumulated in well-defined zones highlighting the focal adhesions of the cells, while after dasatinib and the combined treatments, phospho-paxillin staining was strongly reduced (Figure 3B).

### Dasatinib enhanced RPI-1 effects

Since proliferation was already reduced by 60% in the presence of 100 nM dasatinib, and the phosphotyrosine profiles of the cells treated with the three different dasatinib concentrations did not show significant changes, we used a concentration of 100 nM in all subsequent experiments.

**Figure 4:**
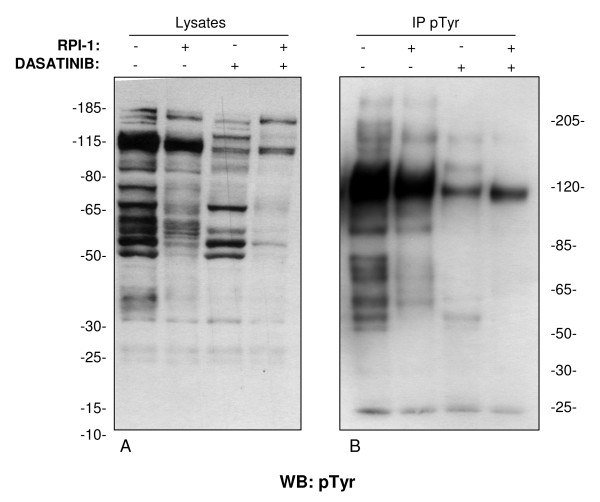
**Phosphotyrosine protein separation of TPC-1 cells before and after drug treatments**. (A) anti-p-Tyr immunoblot of equal amounts of TPC-1 whole-cell extracts from control (0.5% solvent), or cells treated with RPI-1 (40 μM), dasatinib (100 nM), or both, resolved by 4-12% SDS-PAGE. (B) Effects of RPI-1 and dasatinib treatments (used alone or in combination) on the anti-p-Tyr immunoprecipitates from TPC-1 cells, anti-p-Tyr immunostained and resolved by 3-8% SDS-PAGE.

TPC-1 cells were treated with 40 μM RPI-1, 100 nM dasatinib, or with both drugs for 24 hours, then the cells were lysed and extracts were analyzed by immunoblotting (Figure 4). The anti-phosphotyrosine profile of the RPI-1-treated cells differed from that of the dasatinib-treated cells, and both presented a specific reduction in phosphorylated proteins compared with the untreated cells. Notably, only a few bands were prominent in the profile of the combined treatment (Figure 4).

**Figure 5:**
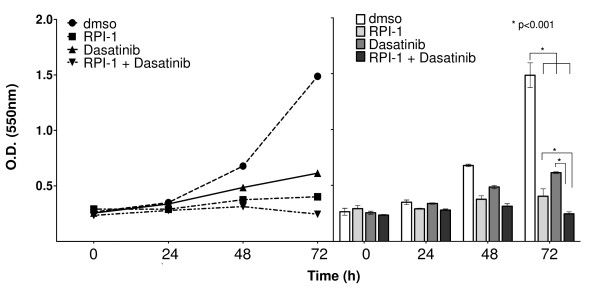
**Proliferation assay**. TPC-1 cells were treated with RPI-1, dasatinib, or both for 24, 48, and 72 hours. After 72 hours, RPI-1 reduced TPC-1 cell proliferation by 70%, while dasatinib treatment resulted in a 60% reduction. The combination of the two drugs reduced the proliferation by 83%. The interaction was significant (p < 0.001).

We also evaluated the effects of the drugs on cell proliferation by performing a sulforhodamine B assay on TPC-1 cells treated for 72 hours. After 24 hours the difference in cell proliferation was not remarkable, but after 72 hours the reduction in cell proliferation was consistent (Figure 5). After 72 hours, RPI-1 reduced TPC-1 cell proliferation by 70%, while dasatinib treatment resulted in a 60% reduction. The combination of the two drugs reduced proliferation by 83%. No evidences of apoptosis was shown (see Additional file 2: PARP immunoblot).

### Characterization of phosphotyrosine profiles and analysis of the RPI-1- and dasatinib-sensitive components in TPC-1 cells

**Figure 6:**
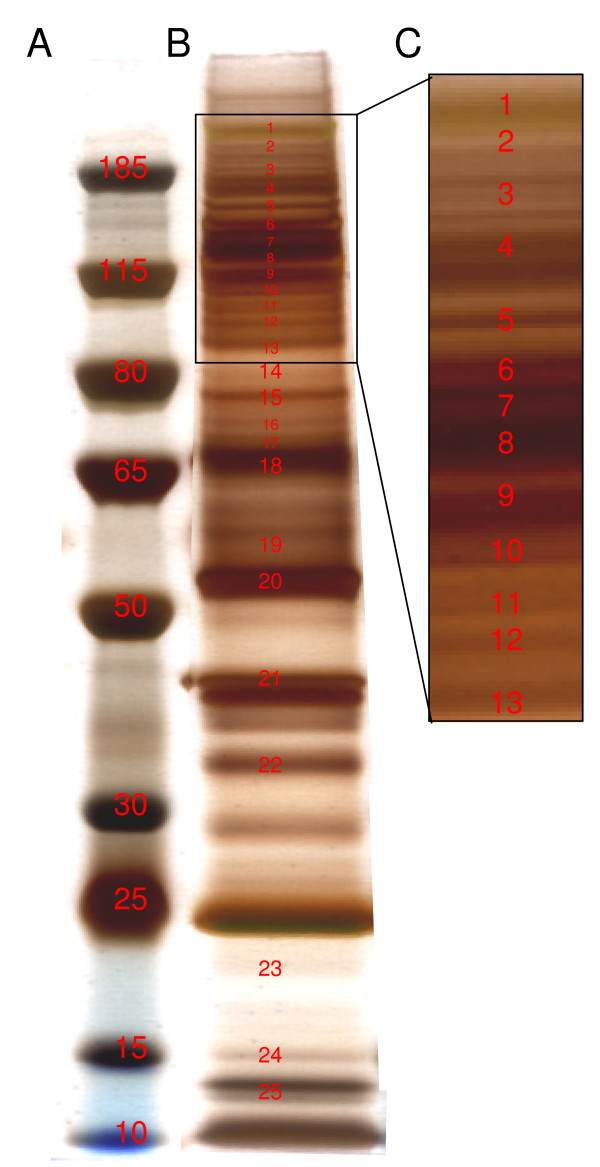
**Identification of most abundant phosphorylated proteins**. (A) Molecular weight markers. (B) Silver staining of anti-p-Tyr affinity-purified proteins. Protein extracts were incubated with anti-p-Tyr agarose-conjugated antibody. Bound proteins were washed, eluted, and resolved by 4-12% SDS-PAGE. Proteins identified are indicated in red and listed in Table 1. (C) Gel zone most abundant in phosphotyrosine proteins.

In order to dissect alternative pathways involved in TPC-1 survival, and to better characterize the effects of the two drugs, we examined the molecular changes induced by dasatinib and/or RPI-1 treatment by analyzing the proteins that were modulated by the drugs. First, we identified the most abundant phosphorylated proteins in untreated TPC-1 cells. Protein bands from untreated TPC-1 lysates were resolved by 4-12% SDS-PAGE, excised from preparative silver-stained gels, and processed for MALDI-TOF mass spectrometry (MS) analysis (Figure 6). The identified proteins are listed in Table 1.

**Table 1:** Immunoaffinity-purified proteins from untreated TPC-1 cells. The band numbers correspond to that of the gel in figure 6

Band	Score	Swiss Prot accession number	entry name	name	Coverage	MW
1	984.72	P35579	MYH9_HUMAN (C_1)	Myosin-9	53	226

	79.00	P35580	MYH10_HUMAN (C_1)	Myosin-10	33	229

2	31.00	Q9HBL0	TENS1_HUMAN (C_1)	Tensin-1	18	186

3	112.96	Q8WWN8	ARAP3_HUMAN (C_1)	Arf-GAP, Rho-GAP domain,	18	170

4	48.02	P00533	EGFR_HUMAN (C_1)	Epidermal growth factor receptor	26	132

5	215.39	P19174	PLCG1_HUMAN (C_1)	1-phosphatidylinositol-4,5-bisphosphate ...	29	149

6	131.26	Q7L576	CYFP1_HUMAN (C_1)	Cytoplasmic FMR1-interacting protein 1	32	145

	43.26	Q96F07	CYFP2_HUMAN (C_1)	Cytoplasmic FMR1-interacting protein 2	20	148

	37.72	P08581	MET_HUMAN (C_1)	Hepatocyte growth factor receptor	20	153

7	192.82	Q7L576	CYFP1_HUMAN (C_1)	Cytoplasmic FMR1-interacting protein 1	34	145

	75.41	P08581	MET_HUMAN (C_1)	Hepatocyte growth factor receptor	26	153

	54.63	Q69YQ0	CYTSA_HUMAN (C_1)	Cytospin-A	28	125

8	34.60	P29597	TYK2_HUMAN (C_1)	Non-receptor tyrosine-protein kinase TYK...	17	134

9	74.45	Q9H5V8	CDCP1_HUMAN (C_1)	CUB domain-containing protein 1	27	90

10	440.36	Q05397	FAK1_HUMAN (C_1)	Focal adhesion kinase 1	46	119

	18.63	Q9Y2A7	NCKP1_HUMAN (C_1)	Nck-associated protein 1	16	129

	24.28	Q9P0K7	RAI14_HUMAN (C_1)	Ankycorbin	21	110

11	38.34	Q96PD2	DCBD2_HUMAN (C_1)	Discoidin, CUB and LCCL domain-containin...	41	78

	29.07	O60716	CTND1_HUMAN (C_1)	Catenin delta-1	24	108

	23.23	P29317	EPHA2_HUMAN (C_1)	Ephrin type-A receptor 2	22	106

12	162.47	Q14764	MVP_HUMAN (C_1)	Major vault protein	42	99

	17.82	O43707	ACTN4_HUMAN (C_1)	Alpha-actinin-4	19	105

13	18.41	O75815	BCAR3_HUMAN (C_1)	Breast cancer anti-estrogen resistance p...	18	93

14	58.87	Q9Y2X7	GIT1_HUMAN (C_1)	ARF GTPase-activating protein GIT1	37	84

	37.01	Q16643	DREB_HUMAN (C_1)	Drebrin	34	71

	21.79	Q13671	RIN1_HUMAN (C_1)	Ras and Rab interactor 1	21	84

15	60.58	Q05655	KPCD_HUMAN (C_1)	Protein kinase C delta type	33	78

16	26.25	P11021	GRP78_HUMAN (C_1)	78 kDa glucose-regulated protein	28	70

17	31.38	P11142	HSP7C_HUMAN (C_1)	Heat shock cognate 71 kDa protein	29	71

18	46.35	Q9H5V8	CDCP1_HUMAN (C_1)	CUB domain-containing protein 1	22	90

19	27.67	Q99704	DOK1_HUMAN (C_1)	Docking protein 1	41	52

20	24.69	Q9BQE3	TBA1C_HUMAN (C_1)	Tubulin alpha-1C chain	27	50

21	135.18	P60709	ACTB_HUMAN (C_1)	Actin, cytoplasmic 1	58	42

	135.18	P63261	ACTG_HUMAN (C_1)	Actin, cytoplasmic 2	58	42

22	47.94	P09493	TPM1_HUMAN (C_1)	Tropomyosin alpha-1 chain	46	33

23	22.66	P19105	MRLC3_HUMAN (C_1)	Myosin regulatory light chain MRLC3	57	20

	22.53	O14950	MRLC2_HUMAN (C_1)	Myosin regulatory light chain MRLC2	57	20

24	47.11	P60660	MYL6_HUMAN (C_1)	Myosin light polypeptide 6	56	17

25	23.73	Q9NYM9	BET1L_HUMAN (C_1)	BET1-like protein	42	12

**Figure 7:**
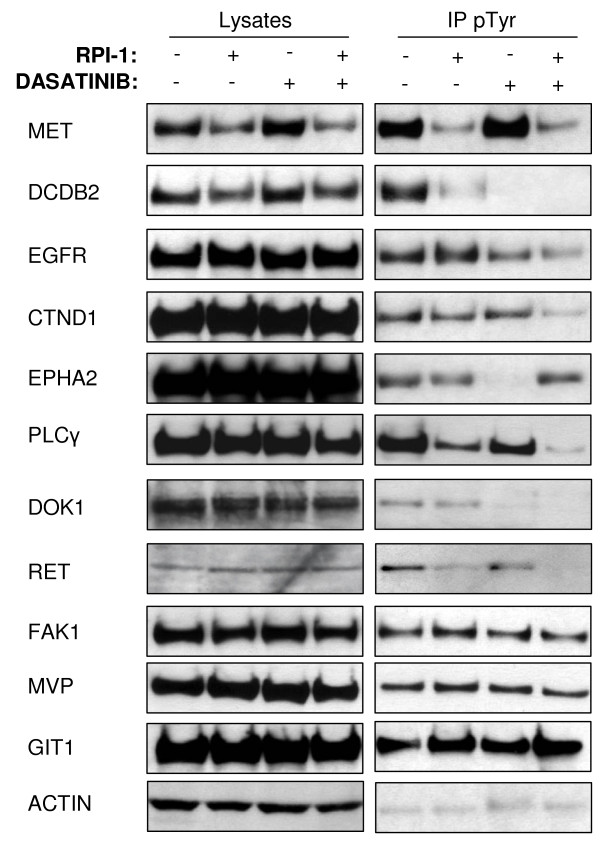
**Validation of identified proteins of interest**. Immunoblot experiments confirming the proteomic selection of proteins sensitive to RPI-1, dasatinib, or both drugs. Anti-p-Tyr immunoaffinity-purified proteins from TPC-1 cells, alone or treated with RPI-1, dasatinib, or both, were resolved by 3-8% SDS-PAGE and immunoblotted with antibodies specific to MET, discoidin (DCDB2), EGFR, CTND1, EPHA2, PLCγ, DOK1, RET/PTC1, FAK1, MVP, and GIT1. Normalization of results was obtained by immunoblotting analysis of beta-actin (ACTB).

**Figure 8:**
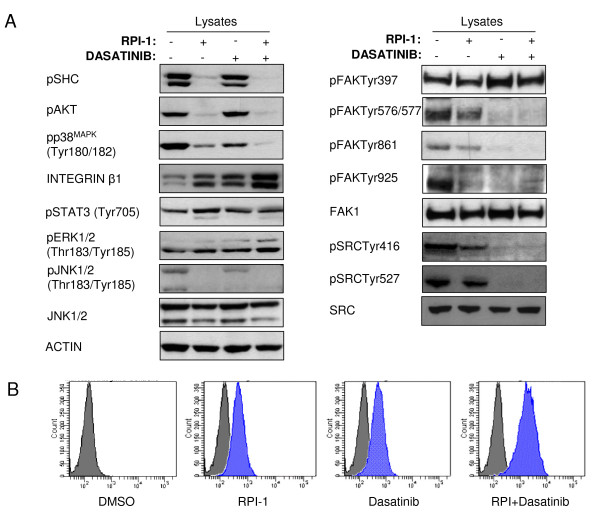
**Immunoblot analysis of the survival pathways**. (A) Whole-cell lysates, untreated or treated with RPI-1, dasatinib, or both, and resolved by 4-12% SDS-PAGE, were analyzed by immunoblot experiments with anti-beta1 integrin and anti-phospho-SHC, -Akt, -p38^MAPK^, -STAT3, -ERK1/2, -FAK, and -Src. (B) FACS analysis of TPC-1 before and after treatments showing an increase in β1-integrin surface expression.

Among the 38 unique proteins identified by MS, we selected 11 proteins as representative of the principal pathways that were analyzed by immunoblotting of anti-phosphotyrosine (p-Tyr) affinity-purified proteins and total lysates from untreated and treated TPC-1 cells (Figure 7). The RPI-1 treatment reduced tyrosine phosphorylation of RET, Met proto-oncogene tyrosine kinase (MET), discoidin (DCDB2), catenin delta-1 (CTND1), and PLCγ. In addition to tyrosine phosphorylation reduction, the total levels of MET and DCDB2 were also reduced (Figure 7). Dasatinib enhanced the reduction in tyrosine phosphorylation of all of the previous proteins, apart from MET, PLCγ and CTND1 (Figure 7). Dasatinib also reduced the phosphorylation of epidermal growth factor receptor (EGFR), ephrin type-A receptor 2 (EphA2), and DOK1. Only three of the 11 proteins remained phosphorylated following the combined treatment (Figure 7). In particular, the tyrosine phosphorylations of GIT1 and the major vault protein (MVP) were unmodified as in the single treatments, while EphA2 phosphorylation, which was strongly decreased by dasatinib alone, increased following the combined treatment (Figure 7). Total level of Focal adhesion kinase 1 (FAK) phosphorylation appeared unmodified in this experimental setting (Figure 7); of note, dasatinib specifically inhibited FAK phosphorylation at residues Y576/577, Y861, and Y925 (Figure 8A).

### Residual survival signaling in TPC-1 cells after treatment with RPI-1 and dasatinib

The combination of the inhibition of RET/PTC by RPI-1 and the inhibition of Src by dasatinib was strongly effective in reducing proliferation showing high cytostatic effects. Therefore, we investigated residual survival signaling in TPC-1 cells by immunoblot analysis of selected survival pathways.

The PI3K/Akt pathway is one of the most important survival pathways. Our data showed that this pathway is inhibited by RPI-1 but not by dasatinib (Figure 8A). In particular, RPI-1 as well as the combination of the two drugs switched off Akt and Shc as previously demonstrated [12].

The RPI-1 and dasatinib treatments increased the amount of ITB1 in a cumulative fashion (Figure 8A/B). To further verify this result, we performed a FACS analysis with anti-ITB1 antibody, revealing a moderate increase in ITB1 surface expression (Figure 8B). Immunostaining experiments reflected a qualitative and diffuse increase in ITB1 expression (see Additional file 3: b1-integrin immunostaining after drug treatments). Since the intracellular integrin signal was mediated principally by activation of FAK, we investigated the phosphorylation status of this kinase. As expected, the phosphorylation of FAK drastically changed after treatment with dasatinib, but not with RPI-1 (Figure 8A). After the inhibition of Src by dasatinib, only the autophosphorylation site (Y397) of FAK remained phosphorylated.

Since it has been shown that activation of ITB1 enhances STAT3-mediated survival signaling, we assayed STAT3 activation. The RPI-1 treatment enhanced the phosphorylated form of STAT3, but dasatinib did not affect it. Regarding the MAP kinase (MAPK) pathway, we found that ERK1/2 activation was slightly increased after pharmacological treatment, p38 phosphorylation was reduced after RPI-1 and dasatinib treatment and switched off after the combination of the two drugs and JNK1/2 phosphorylation was reduced after RPI-1 and the combined treatment (Figure 8A).

## Discussion

In the current study we have investigated the molecular responses of the TPC-1 cell line, spontaneously expressing the RET/PTC1 oncoprotein, toward a combination of two drugs (RPI-1 against RET/PTC1 and dasatinib against Src kinase). All data demonstrate that the combination of the two drugs effectively reduced cell proliferation (by more than 80%) (Figure 5), significantly decreased Tyr phosphorylation of almost all phosphorylable proteins (Figure 4), and altered the morphology of the cells (Figure 2), supporting high cytostatic effects.

The cellular effects of RPI-1 are well known [10-13]; thyroid cell lines treated with this drug strongly maintain FAK activation, allowing them to survive. In order to reduce FAK activation, we treated TPC-1 cells with dasatinib alone or in combination with RPI-1, achieving a consistent reduction in FAK phosphorylation (Figure 8). The treated cells showed an overall reduction of phosphorylated proteins, especially following treatment with dasatinib and the drug combination. Dasatinib alone reduced cell proliferation by 60%, while the combination enhanced the reduction up to 80% (Figure 5). Dasatinib treatment induced morphological changes in TPC-1 cells, resulting in reduction of the phosphorylation levels of cytoskeletal regulators such as Crk and p130^CAS^. Crk is an adaptor protein associated with cell adhesion, and is phosphorylated by SFKs. p130^CAS ^is a docking protein that plays a central role in tyrosine-kinase-based signaling related to cell adhesion, and is phosphorylated by both FAK and SFKs. Inhibition of phosphorylation of these proteins causes an altered maturation of focal complexes to stable focal adhesions, thus reducing lamellipodium formation, migration, and invasion [20,21].

Our paxillin staining experiments (Figure 3B) also suggested altered focal adhesion complex formation. Paxillin is a focal adhesion-associated protein that serves as a docking protein, recruiting signaling molecules to the focal adhesions. Normally, phosphorylated paxillin localizes to focal adhesions, permitting staining of these structures [22]. The expression of a phosphomimetic paxillin mutant not only increased adhesion assembly and turnover, but also stimulated the formation of lamellipodial protrusions [23]. Our findings suggest that, after dasatinib and combined treatments, the phospho-paxillin staining was strongly reduced respect to control cells or cells treated with RPI-1, emphasizing the reduction in focal adhesions produced by dasatinib treatment.

To more deeply understand the molecular effects of the drug treatments, and with the aim of dissecting various pathways involved in TPC-1 proliferation and survival, we analyzed the phosphotyrosine profiles of TPC-1 cells by a proteomic approach, revealing the 38 unique proteins listed in Table 1. As expected, proteins involved in RET signaling, such as MET and PLC-γ, were successfully modulated by RPI-1. Protein kinases including FAK, receptor tyrosine kinases EGFR and EPHA2, and the adhesion molecules discoidin and catenin delta-1 were modulated by dasatinib exposure. Our group of 38 proteins also included components of the cytoskeleton: tropomyosin alpha-1 chain, drebrin, tubulin alpha-1C chain, and others (Table 1). Eleven of the unique proteins were selected as representatives of the most critical functional categories, and drug treatment effects were confirmed by immunoblotting. Eight of these proteins were modulated by the drugs.

Most of the proteins that are modulated by RPI-1 are involved in cell survival and proliferation, including RET, MET, and discoidin. The roles of RET and MET in cancer development and progression are well-known, while the implication of discoidin is a recent finding [24]. In particular, discoidin is involved in cell survival and migration [24], is overexpressed in highly-metastatic lung cancer cells [25], and was shown to be a novel tyrosine phosphorylation substrate of EGF signaling [26].

On the other hand, in agreement with the inhibition of FAK and cytoskeletal proteins, dasatinib preferentially acted on cell adhesion and proliferation by blocking Src kinase. Src interacts with tyrosine-kinase receptors such as EGFR and EPHA2, modulating cell proliferation [27,28], but it is also involved in cell adhesion, as shown by the modulation of cytoskeletal proteins [20,21]. In breast cancer, Src interacts with EGFR, enhancing the activation of mitogenic signaling and promoting cancer progression [27]. Dasatinib inhibits growth of breast cancer cells by modulating EGFR signaling [28]. Here the inactivation of Src reduced the phosphorylation of EGFR, suggesting an interaction between Src and EGFR (Figure 7). DOK1, a negative regulator of the Ras-Erk pathway [29], is another dasatinib-modulated protein that was shown to interact with a broad range of signaling proteins implicated in the regulation of physiological responses, including negative regulation of cytokines, enhanced cell migration, and filopodia extension [30]. In our study, DOK1 tyrosine phosphorylation did not seem to be dependent on RET/PTC1 (Figure 7), in contrast with DOK1 function in medullary thyroid carcinoma, where its phosphorylation was mediated by mutated RET [31]. In fact, DOK1 tyrosine phosphorylation was reduced exclusively after dasatinib treatment (Figure 7). The behavior of EphA2 phosphorylation was intriguing. EphA2 is an RTK involved in cancer development and progression, especially when in its non-phosphorylated state [32]. Non-phosphorylated EphA2 associates with FAK and induces the auto-phosphorylation of FAK Y397 that sustains integrin activation [32]. However, phosphorylated EphA2 stimulates cell survival and migration [33]. In our study, EphA2 decreased its phosphorylation after dasatinib treatment, but after the combined treatment it conserved its phosphorylation (Figure 7). Thus, we suggest that EphA2 contributes to survival and migration via FAK in dasatinib-treated cells, but functions independently of FAK in RPI-1+dasatinib-treated cells.

**Figure 9:**
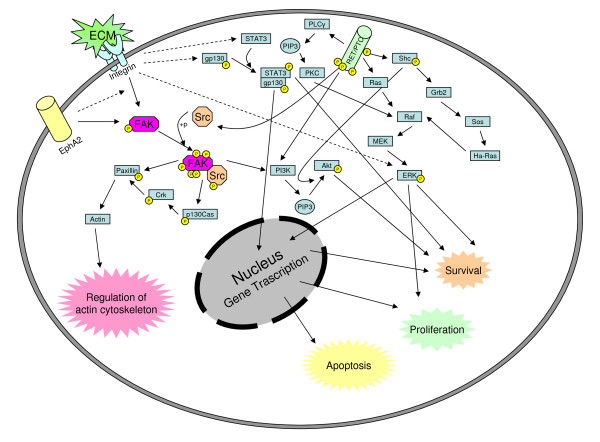
**Principal pathways involved in TPC-1 cell survival and proliferation**. Schematic representation of the principal pathways involved in TPC-1 cell survival and proliferation. RPI-1 acts on RET/PTC, blocking phosphorylation of the downstream proteins Akt and Shc. Dasatinib acts on Src, causing changes in focal adhesion proteins and proliferation. After treatment with the combination of RPI-1 and dasatinib, STAT3 and ERK1/2 remained active, possibly contributing to cell survival.

Finally, we wished to understand how some cells survived following the combined treatments, by investigating survival pathways (Figure 9). The increase in ITB1 after dasatinib treatment suggests that survival may be mediated by adhesion molecules. Since the intracellular integrin signal was mediated principally by activation of FAK, we investigated its phosphorylation status. As expected, the phosphorylation status of FAK drastically changed after treatment with dasatinib, but not with RPI-1, and the autophosphorylation site of FAK (Y397) remained phosphorylated (Figure 8A).

PI3K and Shc interact with phospho-Y397 of FAK, leading to activation of pro-survival proteins such as Akt. Following treatment with the drug combination, Akt phosphorylation was completely abrogated while, after dasatinib alone, Akt activation was not affected, confirming that Akt activation is driven by RET and not by the FAK/Src complex (Figures 8, 9) [12,14].

Recent work by Shain et al. [34] has demonstrated that the fibronectin (FN)-mediated activation of ITB1 enhances the STAT3-mediated survival signaling in myeloma cells, conferring resistance to apoptosis. RPI-1 treatment enhanced the phosphorylation form of STAT3, but dasatinib did not affect it (Figure 8). STAT3 is over-phosphorylated in most cancers, preventing apoptosis and enhancing proliferation, angiogenesis, and invasion [35]. Here, STAT3 remained phosphorylated after the combination of the two drugs (Figure 8A), probably contributing to cell survival.

The MAPK pathway regulates various cellular activities, and is deregulated in one-third of all cancers. The effects of ERK activation vary by cell type, and impact diverse cellular processes such as proliferation, differentiation, survival, migration, and angiogenesis. p38 negatively regulates cell proliferation and tumorigenesis via its pro-apoptotic functions. Recent studies have shown that p38 initiates apoptosis after oxidative stress induced by activated Ras [36], antagonizing the oncogenic activity of Ras. p38 activation is essential for the antileukemic effects of dasatinib [37]. JNK can exert dual functions, which range from the induction of apoptosis to increased survival by modulating both phosphorylation and expression of several members of the Bcl-2 protein family [38]. Consequently, we investigated the MAPKs in our system, finding that ERK1/2 was slightly increased after pharmacological treatments, while p38 and JNK1/2 was switched off. Transfection of ITB1 into hepatocarcinoma cells protects them from apoptosis induced by chemotherapeutic drugs, by activating the MAPK pathway [39]. ERK activation could be enhanced as a secondary effect of dasatinib treatment inactivating the negative regulators DOK1 and p38 [29,36].

To further confirm the hypothesis that survival could be mediated by ITB1, we combined the pharmacological treatments with two different integrins inhibitors, a blocking antibody and a RGD-mimic pentapeptide, but the inhibition in proliferation was not statistically significant compared with the inhibition achieved by the combination of the two drugs (data not shown). A possible explanation for this behavior could be an abundance of extracellular matrix proteins, such as fibronectin (data not shown), and adhesion molecules, for instance SPARC produced by TPC-1 cells, that could antagonize integrin inhibition. SPARC can modulate ERK activation by activating a G-protein coupled receptor, converging on the integrin signaling pathway [40], as well as EphA2 receptor known for its importance in cancer development and its ability to converge on integrin signaling [32,33].

## Conclusions

The current study suggests that RPI-1 and dasatinib act on two different pathways; dasatinib acts on the FAK/Src pathway, while RPI-1 heavily impairs the tyrosine kinase activity of RET. Following a combined treatment, cell survival pathways appeared to be mediated by STAT3 and ERK activities resulting from integrin clustering and FAK autophosphorylation (Figure 9) [41,42]. EphA2 may also contribute, at least in part, to integrin and FAK activation [32,33]. Our data are in agreement with current evidence that cell adhesion-mediated drug resistance is an important mechanism that may enable tumor cell survival, bringing resistance to drug-induced apoptosis [34,43]. In conclusion, dasatinib, in combination with RPI-1, strongly reduced proliferation, altered morphology, and changed the phosphorylation protein profile of TPC-1 cells, implicating ITB1 and EphA2 as promising therapeutic targets in PTC.

## Methods

### Cell Culture and Drug Treatment

TPC-1 cells were grown in Dulbecco's modified Eagle's medium (DMEM, GIBCO, Carlsbad, CA, USA) supplemented with 10% fetal bovine serum FBS (HyClone Laboratories, Logan, UT, USA) and 1 mM sodium pyruvate. For cell treatment, RPI-1 (8 mM in 100% dimethylsulfoxide, DMSO) and dasatinib (0.2 mM in 100% DMSO) were directly diluted in cell culture medium to achieve a final concentration of 40 μM for RPI-1 and 100 nM for dasatinib. The final solvent concentration was less than 0.5% for all samples (including controls).

### Proliferation assay and immunofluorescence

The sulforhodamine B (SRB) proliferation assay was used for cell density determination, based on the measurement of cellular protein. SRB was performed in 96 multiwell plates containing cells, seeded at 2000 cell/cm^2 ^for 18 h, treated with drugs and fixed with TCA 50% in ddH_2_O. Fixed cells were stained with 0.4% (wt/vol) SRB solution for 30 min and rinsed with 1% (vol/vol) acetic acid. Protein-bound dye was solubilized with 10 mM Tris base (pH 10.5) and the optical density at 550 nm was determined.

For immunofluorescence, cells were grown and treated on glass chamber slides, fixed in 4% paraformaldehyde and sucrose, permeabilized with 1% BSA and 0.1% Triton X-100, saturated with 10% goat serum, and stained with fluorescent phalloidin (Sigma Aldrich, St. Louis, Missouri, USA), paxillin (Transduction Laboratory, BD, Franklin Lake, NJ, USA), and DRAQ5 (Biostatus Limited, LE, UK) as nuclear markers. Slides were prepared using ProLong Antifade mounting media (Invitrogen S.r.l, MI, IT), and imaged with confocal microscopy (Microradiance 2000; Bio-Rad Laboratories) equipped with argon/krypton (488 nm), HeNe (543 nm), and red laser diode (638) lasers. Confocal images (512 × 512 pixels) were obtained using a 60× oil immersion lens and were analyzed using ImagePro 6.3 software. Reported images represent extended depth of field from 10-15 frames in a stack (0.5 μm step); focus regions were selected for maximum intensity. The pinhole diameter was regulated according to the value suggested by the acquisition software manufacturer to obtain the maximum resolution power.

### FACS analysis

Cells were seeded on 100-mm tissue culture plates, incubated for 18 h, then treated with drugs for 24 h and then washed with PBS, pelleted at 800 rpm for 5 min, and resuspended at a concentration of 1×10^6 ^cells/mL in PBS. After blocking with normal goat serum for 30 minutes, cells were incubated with anti-β1 integrin antibody (Clone MAR6) kindly provided by Dr.ssa Tagliabue E for 1 h at room temperature (RT), pelleted, and washed three times with PBS to remove excess primary antibody. Cells were then resuspended in 1 ml of PBS and incubated with Alexa Fluor(r) 555 (goat anti-mouse IgG, red) fluorescent labeled secondary antibody for 30 min at RT. After three more washes, cell pellet was resuspended in PBS and analyzed on a FACsDIVA (Becton Dickinson).

### Sample preparation and Anti-p-Tyr immunoprecipitation

TPC-1 cells were seeded at 3 × 10^4 ^cells/cm^2^, cultured for 18 h, and exposed to the drugs for 24 h before cell lysis. Treated and control cells were washed 5 times with cold phosphate-buffered saline (PBS) containing 0.1 mM sodium orthovanadate, and harvested by scraping into another 0.5 ml cold PBS before centrifugation at 2000 × g for 5 min at 4°C. The supernatants were discarded and the cell pellets were solubilized in cold lysis buffer containing 50 mM HEPES (pH 7.6), 150 mM NaCl, 10% glycerol, 1% Triton X-100, 1.5 mM MgCl_2_, 1 mM EGTA, 10 mM Na_4_P_2_O_7_, 100 mM NaF, and 1 mM sodium orthovanadate in the presence of protease inhibitors (Sigma Aldrich, St. Louis, MO, USA). After 30 min of incubation with gentle rocking at 4°C, lysates were cleared by centrifugation. Supernatants were collected and protein concentrations were determined by Bradford or BCA assays (Bio-Rad Laboratories Srl, MI, IT).

For anti-p-Tyr immunoprecipitation, samples were processed as previously described [13].

### SDS-PAGE and Immunoblotting

All electrophoresis (SDS-PAGE) and electroblotting experiments were performed with precast polyacrylamide NuPAGE NOVEX gels (Invitrogen, MI, IT), and with Hybond-C super nitrocellulose membrane (Amersham Biosciences, Little Chalfont, UK). For immunoprecipitation experiments, equal amounts of protein from treated cell lysates (30 μg) and immunoprecipitates were compared with non-treated samples on 3-8% precast gels and processed as previous described [13]. Proteins were transferred to nitrocellulose membranes and probed with the appropriate antibodies. Immunoreactive bands were visualized using horseradish peroxidase-linked anti-mouse, anti-rabbit, or anti-goat antiserum and detected using an enhanced ECL system (Bio-Rad Laboratories Srl, MI, IT).

### In-gel tryptic digestion, MALDI-TOF MS, and peptide mass fingerprinting

For MALDI-TOF identification, anti-pTyr immunoprecipitated proteins were resolved by SDS-PAGE, followed by silver or colloidal Coomassie staining, according to standard procedures. Protein bands were excised and processed as previously described [13]. Briefly, reduced and carbamylated proteins were in-gel digested with trypsin (13 ng/μl) for 18 h at 37°C. The peptide mixture obtained from each band was analyzed by MALDI-TOF Voyager-DE STR (Applied Biosystems, Framingham, MA, USA), equipped with a nitrogen laser (337 nm). The resulting spectra were analyzed with Aldente software (http://www.expasy.ch/tools/aldente/). Input was searched according to the following database: Aldente: UniProtKB/SwissProt; predefined taxon: Mammalia; Spectrometer internal error max: 25. Only proteins identified in at least three separate experiments were considered.

### Antibodies and reagents

For immunoprecipitation experiments, anti-phosphotyrosine agarose-conjugated PT66 (anti-p-Tyr, Sigma-Aldrich) was used. For immunoblotting, the following antibodies were used: anti-p-Tyr 4G10, anti-pShc, anti-Src (Upstate Biotechnology, Billerica, MA, USA), anti-discoidin, anti-p130^CAS ^(Abcam, Inc., Cambridge, MA, USA), anti-RET (C-19), anti-EGFR (1005), anti-GIT1 (H170), anti-EPHA2 (H175), anti-MET (C12), anti-PLCγ1 (1249), anti-β1-INTEGRIN (N-20), anti-pFAK (Y576/577, Y861, and Y925), anti-DOK1 (Santa Cruz Biotechnology, Santa Cruz, CA, USA); anti-FAK1, anti-p130^CAS^, anti-paxillin, anti-Crk (Transduction Laboratory, BD, Franklin Lake, NJ, USA); anti-beta-actin; anti-catenin delta-1 (Histo-Line Laboratories, Milan, Italy), anti-MVP (US Biological, Swampscott, MA, USA), pp38^MAPK ^(Y180/182), anti-pSTAT3 (Y705), anti-pAkt (Ser 473), anti-pSrc (Y527 and Y416), anti-pFAK (Y397), anti-pPaxillin (Y118), anti-pp130^CAS ^(Y410) (Cell Signaling Technology, Inc., Boston, MA, USA), anti-pERK1/2 (T183/Y185) (Sigma Aldrich, St. Louis, MO, USA).

Tris, SDS, HEPES, glycerol, Triton X-100, NaCl, and DMSO were obtained from Sigma Fluka (St. Louis, MO, USA), silver stain reagents were obtained from Sigma Aldrich. Coomassie Brilliant Blue G250 reagent stain was obtained from Bio-Rad.

### Statistical analysis

Each experiment was performed at least twice. The statistical significance of the results was determined using Student's t-test. Data were considered significant when p ≤ 0.05.

## Competing interests

The authors declare that they have no competing interests.

## Authors' contributions

Conceived and designed the experiments: DC and IB; Performed the experiments: DC, FM, PM, PC and GC; Analyzed the data and provided the interpretations: DC and IB; Wrote the manuscript: DC and IB; Critically revised the manuscript: GC; All authors read and approved the final manuscript.

## Supplementary Material

Additional file 1 **Immunoblot analysis of p130^CAS ^immunoprecipitation**. Whole cell lysates (WCL) of TPC-1 before and after drug treatments were immunoprecipitated with anti-p130^CAS ^antibody (Abcam, Inc., Cambridge, MA, USA). Reductions in both p130^CAS ^protein and its phosphorylation in the immunodepleted (ID) samples were observed. The absence of the p130^CAS ^protein in the two pre-cleared (PC) lysates highlighted the specificity of the immunoprecipitation. The positive control (Ctr +) was the WCL of untreated cells. Click here for file

Additional file 2 **PARP immunoblot**. TPC-1 lysates before and after drug treatments were stained for the C-terminal domain of the PARP protein, a marker of cell apoptosis, using an anti-PARP antibody (Cell Signaling Technology, Inc., Boston, MA, USA). During apoptosis, activated caspase-3 cleaves PARP protein (116 kDa) into an N-terminal domain (24 kDa) and a C-terminal domain (89 kDa). The total amount of full-length PARP (116 kDa) was not modified after the treatments, suggesting the absence of apoptosis. Click here for file

Additional file 3 **β1-integrin immunostaining after drug treatments**. Immunofluorescence microscopy of TPC-1 cells before and after drug treatments. Cells were stained with anti-b1-integrin antibody (kindly provided by Tagliabue E) (red) and DRAQ5 (blue). The staining revealed a qualitative increase in b1-integrin staining, in agreement with the biochemical and FACS analyses (Figure 8A). Images (512 × 512 pixels) were obtained using a 60× oil immersion lens and were analyzed using ImagePro 6.3 software. Scale bars, 10 μm. Click here for file
