# Supplementary figures and images for: Dasatinib reduces FAK phosphorylation increasing the effects of RPI-1 inhibition in a RET/PTC1-expressing cell line
Source: Mol Cancer. 2010 Oct 18;9:278. doi: 10.1186/1476-4598-9-278 (PMC2967544; doi:10.1186/1476-4598-9-278)

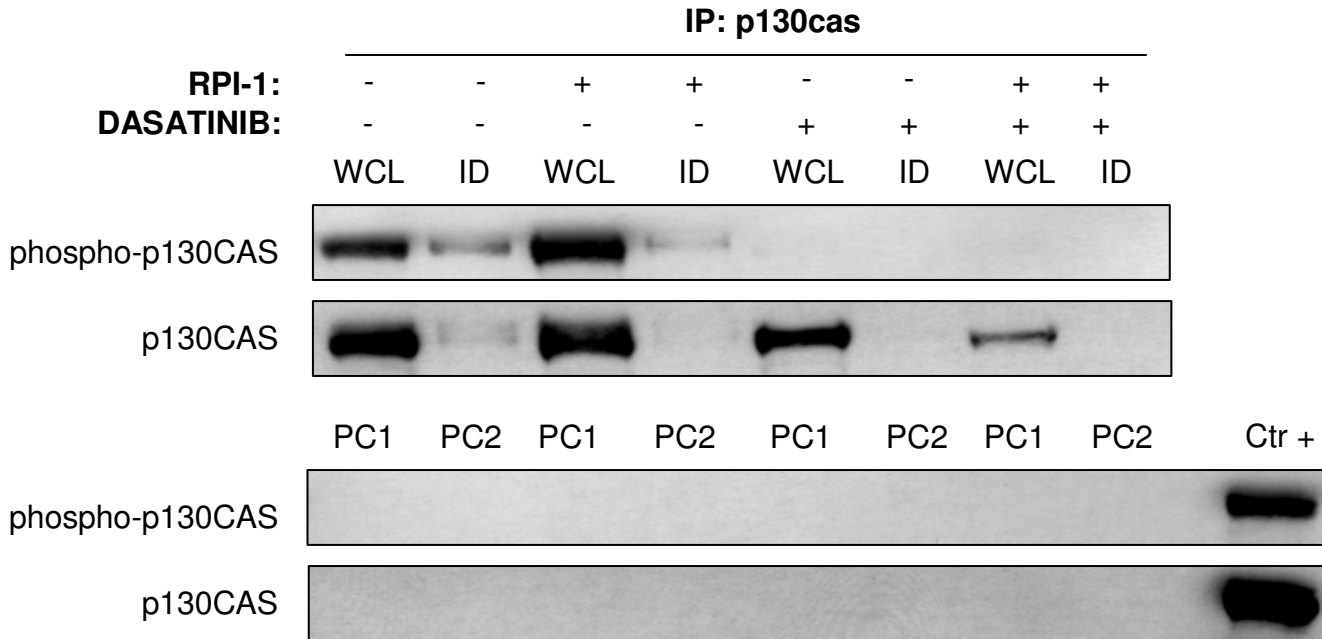

Supplement: Additional file 1 — Immunoblot analysis of p130CAS immunoprecipitation. Whole cell lysates (WCL) of TPC-1 before and after drug treatments were immunoprecipitated with anti-p130CAS antibody (Abcam, Inc., Cambridge, MA, USA). Reductions in both p130CAS protein and its phosphorylation in the immunodepleted (ID) samples were observed. The absence of the p130CAS protein in the two pre-cleared (PC) lysates highlighted the specificity of the immunoprecipitation. The positive control (Ctr +) was the WCL of untreated cells. [file 1476-4598-9-278-S1.PDF]

|                   | Lysates |   |   |   |
|-------------------|---------|---|---|---|
| <b>RPI-1:</b>     | -       | + | - | + |
| <b>DASATINIB:</b> | -       | - | + | + |

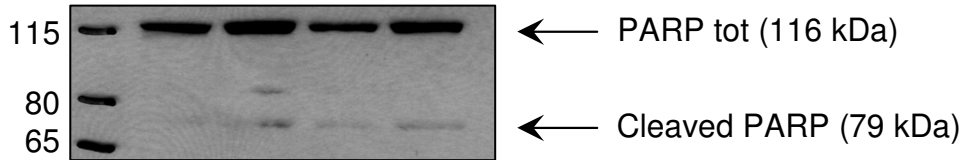

Supplement: Additional file 2 — PARP immunoblot. TPC-1 lysates before and after drug treatments were stained for the C-terminal domain of the PARP protein, a marker of cell apoptosis, using an anti-PARP antibody (Cell Signaling Technology, Inc., Boston, MA, USA). During apoptosis, activated caspase-3 cleaves PARP protein (116 kDa) into an N-terminal domain (24 kDa) and a C-terminal domain (89 kDa). The total amount of full-length PARP (116 kDa) was not modified after the treatments, suggesting the absence of apoptosis. [file 1476-4598-9-278-S2.PDF]

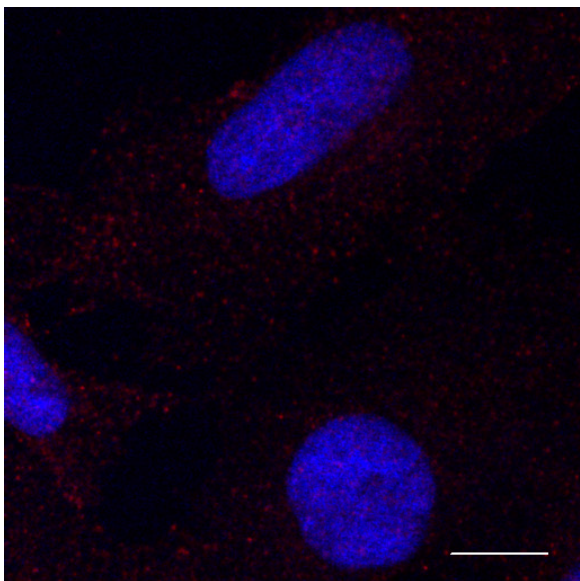

**DMSO**

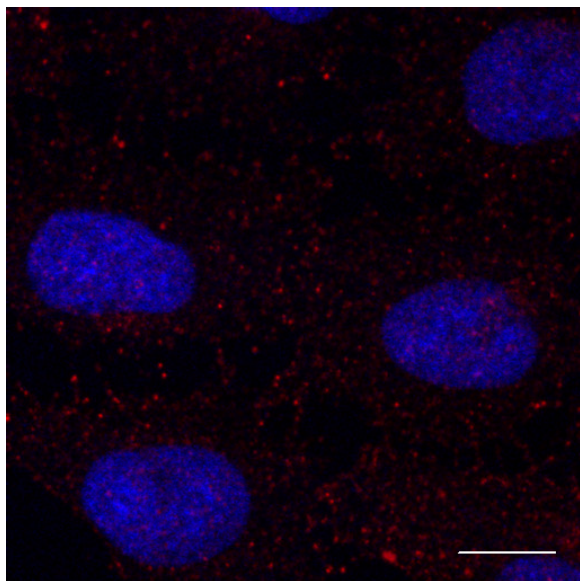

**RPI-1**

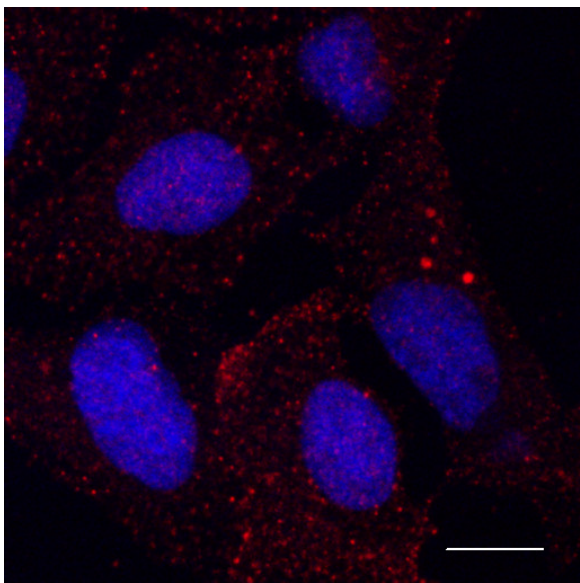

**Dasatinib**

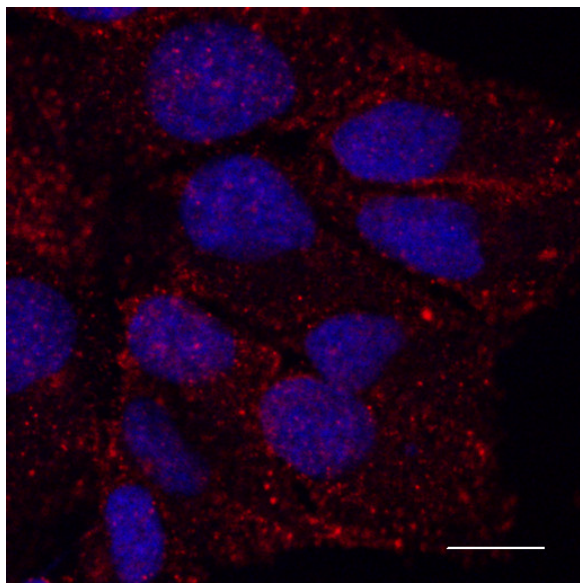

**RPI-1 + Dasatinib**

Supplement: Additional file 3 — β1-integrin immunostaining after drug treatments. Immunofluorescence microscopy of TPC-1 cells before and after drug treatments. Cells were stained with anti-b1-integrin antibody (kindly provided by Tagliabue E) (red) and DRAQ5 (blue). The staining revealed a qualitative increase in b1-integrin staining, in agreement with the biochemical and FACS analyses (Figure 8A). Images (512 × 512 pixels) were obtained using a 60× oil immersion lens and were analyzed using ImagePro 6.3 software. Scale bars, 10 μm. [file 1476-4598-9-278-S3.PDF]
